# Complete mitochondrial genome of *Aethia cristatella* (Charadriiformes: Alcidae)

**DOI:** 10.1080/23802359.2019.1693285

**Published:** 2019-12-09

**Authors:** Jung A Kim, Seung-Gu Kang, Jin Won Yang, Wee-Haeng Hur, Hyun-Jong Kil

**Affiliations:** a Animal Resources Division, National Institute of Biological Resources, Incheon, Republic of Korea;; b Busan Wild Animal Rescue Center, Busan, Republic of Korea

**Keywords:** *Aethia cristatella*, crested auklet, mitochondrial genome, Alcidae

## Abstract

The complete mitochondrial genome sequence of the crested auklet, *Aethia cristatella*, was obtained using high-throughput whole genome sequencing. This is the first report indicating that the complete mitochondrial genome of *Aethia* has been sequenced. The circular genome is 16,848 bp in length. It contains thirteen protein-coding genes, twenty-two transfer RNAs, two ribosomal RNAs, and a control region. The *ND3* gene possessed an insertion mutation. Maximum likelihood phylogenetic analysis demonstrated that *A. cristatella* is the sister clade of *P. aleuticus* clustered with the Alcinae species, belonging to Alcidae.

The crested auklet (*Aethia cristatella*) is the first record in Korea (Yang et al. 2019). *A. cristatella* is a single subspecies belonging to the family Alcidae (Clements 2000; del Hoyo and Collar 2014). The recorded breeding area is the northwest Pacific region including the Bering Sea islands, Aleutians, the Okhotsk Sea, and uninhabited islands. The crested auklet can be found further south in the winter. Certain groups move to Hokkaido and northern Honshu of Japan through post-breeding dispersion (Brazil 2009; del Hoyo and Collar 2014). Globally, the population is decreasing due to competition with allied species or environmental pollution. However, the species of interest, is classified as Least Concern (LC), as the decrease is not sharp and the population is considered stable (IUCN 2016).

In this study, we used Illumina Hiseq to sequence the complete mitochondrial genome of *A. cristatella*. Using the sequencing result and publicly available GenBank data, we confirmed the phylogenetic relationships among the family Lari in the order Charadriiformes.

Samples from *A. cristatella* (IN4608) were discovered by the local Wild Animal Rescue Center on the beach (35˚04′01.31″N, 129˚00′30.53″N) in Seo-gu, Busan, South Korea, and deposited in the National Institute of Biological Resources(NIBR) at Inchoen, South Korea. Genomic DNA (IN4608) was extracted from tissue using a DNeasy Blood and Tissue Kit (Qiagen, Valencia, CA, USA), in accordance with the manufacturer’s protocol and deposited in the NIBR. DNA Link (Seoul, South Korea) performed whole genome resequencing with the Illumina HiSeq 2500 platform. DOGMA (Wyman et al. 2004) and ARWEN (Laslett and Canback 2008) were used for annotation.

The total length of the *A. cristatella* (GenBank accession: MN337912) mitochondrial genome is 16,848 bp, with a 54.9% A + T base composition. The mitochondrial genome is comprised of thirteen protein-coding genes, twenty-two transfer RNAs, two ribosomal RNAs, and a control region. Similar to several avian species (Mindell et al. 1998), an extra A nucleotide at the 175 site (insertion mutation) is present in the *ND3* gene.

We constructed a neighbor-joining tree using MEGA6.0 (Arizona State University, USA), using the thirteen protein-coding genes and two ribosomal RNA genes sequenced here, along with twenty-one Charadriiformes mitochondrial genomes, downloaded from GenBank (Figure 1). Phylogenetic analysis confirmed the monophyly of Fraterculinae. The former subfamily clustered closely with Alcinae, belonging to Alcidae. In conclusion, this study provides a new addition to the avian mitochondrial genome database, thus enriching in evolutionary and ecological studies.

**Figure 1.**
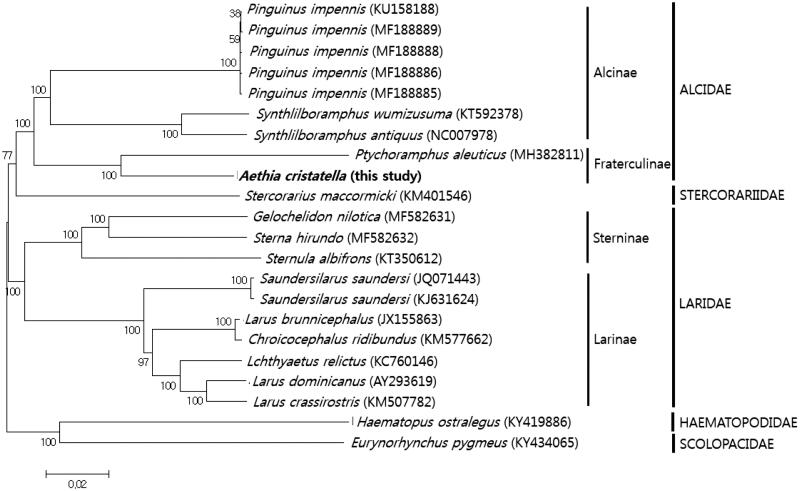
Phylogenetic relationships among 23 Lari groups in Charadriiformes mitochondrial genomes. The numbers present on the nodes indicate percentages of 1000 bootstrap values, estimated for concatenated sequences of 13 protein-coding genes and two ribosomal RNA genes.
